# Evaluation of Prompt Design and Internal Reasoning in Chatbot-Based Medical History Taking: Simulation Study

**DOI:** 10.2196/94614

**Published:** 2026-08-21

**Authors:** Nattawipa Thawinwisan, Chang Liu, Goshiro Yamamoto, Kazumasa Kishimoto, Yukiko Mori, Tomohiro Kuroda

**Affiliations:** 1 Graduate School of Medicine, Kyoto University, 54 Shogoin-kawahara-cho, Sakyo-ku, Kyoto, Japan, 81 75-366-7701; 2 Preemptive Medicine and Lifestyle-Related Disease Research Center, Kyoto University Hospital, Kyoto, Japan; 3 Division of Medical Information Technology and Administration Planning, Kyoto University Hospital, Kyoto, Japan

**Keywords:** chatbots, medical history taking, large language models, prompt engineering, clinical documentation, preconsultation assessment

## Abstract

**Background:**

A persistent discrepancy exists between patient-reported information and physician documentation. While conversational agents have been developed to collect medical histories prior to consultations, existing evaluations have largely focused on diagnostic accuracy or user satisfaction rather than on the completeness and clinical relevance of the information collected. There remains a need to assess the extent to which clinically relevant information is captured through chatbot-based interviews, and to understand how model configurations and instructional strategies influence this coverage.

**Objective:**

This study aimed to evaluate the extent to which a chatbot can obtain clinically useful patient history information, and to examine how prompt detail and internal reasoning influence information coverage during chatbot-based medical interviews.

**Methods:**

We developed a medical history-taking chatbot using the Qwen3-14B-Instruct model and evaluated 4 configurations in a 2×2 factorial design: detailed and thinking mode, detailed and nonthinking mode, minimal and thinking mode, and minimal and nonthinking mode. These configurations were compared against a rule-based system baseline (choice mode) using 66 standardized primary care clinical cases, with simulated patients interacting with the chatbot according to predefined case scripts. Information coverage (%) was assessed using a checklist inspired by Objective Structured Clinical Examination (OSCE) frameworks. Three physicians independently evaluated transcript coverage, with interrater agreement assessed using full agreement rates and Fleiss κ. For the 4 LLM configurations, coverage was analyzed using 2-way repeated-measures ANOVA to examine the effects of prompt detail, internal reasoning, and their interaction. All 5 configurations, including the rule-based baseline, were additionally compared using 1-way repeated-measures ANOVA with post hoc 2-tailed paired *t* tests.

**Results:**

Interrater agreement was substantial (Fleiss κ=0.75). Across all 66 simulated cases, information coverage differed significantly among configurations (*P*<.001), with the detailed prompt with thinking (detailed and thinking) mode achieving the highest mean coverage (72.3%, SD 14.3%), compared with moderate coverage in configurations using either thinking or detailed prompts alone (approximately 60%) and lower coverage in minimal nonthinking and rule-based configurations (approximately 51%‐54%). In the factorial analysis of the 4 LLM configurations, both prompt detail and internal reasoning were significantly associated with improved information coverage, with a significant interaction between prompt detail and internal reasoning interaction. Mode-related differences were most pronounced for past medical and family history domains. Symptom-level analyses revealed substantial variability, with higher coverage for symptoms associated with well-defined diagnostic frameworks and lower coverage for multisystem presentations.

**Conclusions:**

In this controlled, simulated setting, the detailed prompt with a thinking mode achieved the highest overall checklist-based information coverage. The findings suggest that combining structured clinical prompts with internal reasoning may improve the completeness of chatbot-collected patient histories. Further research is needed to evaluate its impact on clinical documentation, workflow integration, and real-world usefulness.

## Introduction

Information about medical history and personal perceptions provided by patients is essential for effective clinical care [1-4]. However, studies have consistently demonstrated a persistent discrepancy between what patients report and what physicians document during clinical encounters [5-8]. This discrepancy often arises from time constraints, selective note-taking, and different communication styles [8]. The absence of this information can potentially hinder accurate diagnoses, continuity of care, and patient-centered decision-making.

To bridge these informational gaps, various digital tools have been developed to collect patient data before consultation [9]. These systems aim to improve the completeness and efficiency of clinical documentation. Studies have shown that computerized history-taking enhances data quality and consistency compared to physician-recorded histories alone [10-13]. More recently, conversational agents or chatbots have emerged as promising tools in health care [14,15]. Evidence suggests that chatbots can capture clinically relevant information while maintaining patient engagement and satisfaction, offering continuous and automated data collection that has the potential to improve the completeness and accessibility of medical history-taking [16].

Despite the growing accessibility of these technologies, physicians’ acceptance of AI tools remains limited due to concerns about transparency, interpretability, and accountability [17,18]. These concerns underscore the importance of evaluating AI-based tools using clinically interpretable outcome measures, rather than relying solely on technical performance metrics.

Existing preconsultation or history-taking tools are usually choice-based and structured [9,19,20]. Although these systems are interpretable and support standardized documentation, their predefined options may constrain the capture of individualized or nuanced patient information. These limitations highlight the need for more flexible and adaptive approaches that maintain interpretability while capturing richer patient narratives.

Recent advances in large language models (LLMs) have significantly enhanced their contextual understanding and multistep reasoning capabilities, facilitating more coherent and clinically relevant patient interactions. Modern LLMs can now use an internal reasoning process, frequently termed “thinking,” to decompose complex clinical prompts into a sequence of intermediate logical steps before generating a final response. This deliberative process has been shown to improve performance on multifaceted tasks, leading to the generation of more structured and clinically aligned medical inquiries [21,22]. However, their outputs depend on prompt design, which is critical for guiding model behavior and reasoning structure [23-25]. Carefully constructing prompts to follow clinical reasoning frameworks can improve history coverage, mitigate premature diagnostic closure, and make the model’s underlying logic more transparent.

Evaluations of AI-based conversational agents remain limited and have primarily focused on diagnostic accuracy, report quality, and user experience rather than on the completeness or clinical relevance of the information collected [26].

Detailed patient histories contribute to accurate diagnoses, treatment plans, and follow-ups by enabling physicians to systematically reason through symptom onset, progression, and contextual factors [27]. When AI-assisted systems gather more complete information, they provide richer data for clinical decision-making and are less likely to prematurely close or jump to conclusions without sufficient information. Evaluating AI tools based on detail and reasoning coverage provides additional clinically useful aspects beyond diagnosis or satisfaction alone.

To address this need, we adopted an evaluation inspired by the Objective Structured Clinical Examination (OSCE) framework, which is widely used and trusted by physicians to assess clinical competence [28]. OSCEs use checklist-based assessments to measure specific skills, ensuring interpretability and transparency. Our study applies a similar, checklist-driven approach, developed from representative clinical cases, to evaluate the coverage and completeness of AI-assisted history taking in a structured and clinically interpretable manner. This study aims (1) to assess the extent to which the chatbot can obtain clinically useful information, as measured by coverage of a case-based history-taking checklist, and (2) to determine the effects of internal reasoning and prompt detail on the chatbot’s performance in achieving comprehensive history coverage.

## Methods

### Chatbot

#### Chatbot Creation

**Figure 1.**
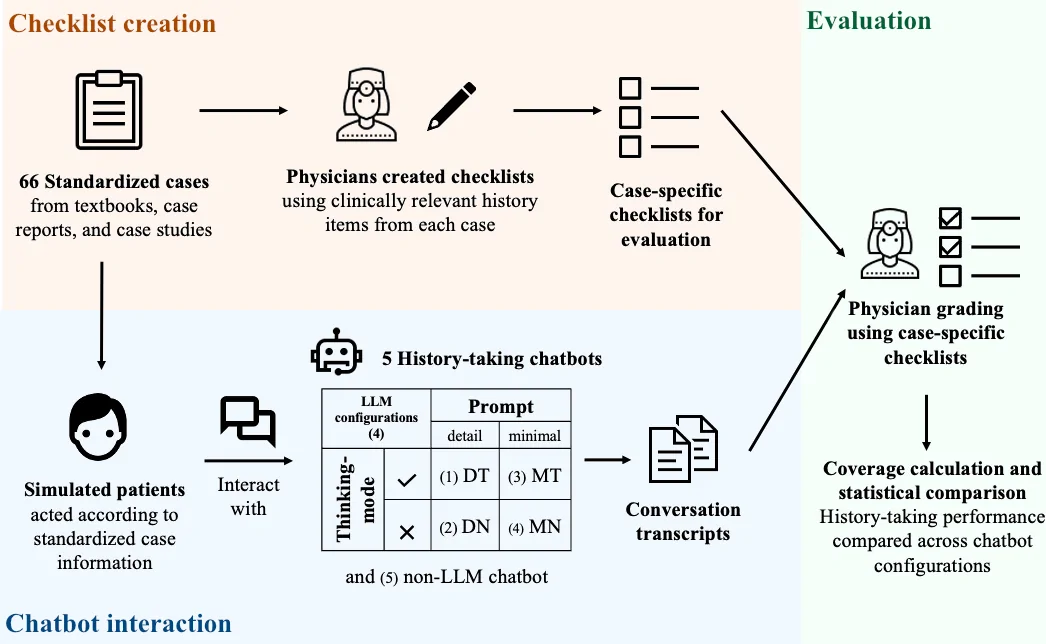
Experimental flow: standardized clinical cases (n=66) were compiled from medical textbooks, case reports, and case studies. Physicians extracted clinically relevant history items from each case to create case-specific evaluation checklists. Simulated patients reviewed the case scenarios and interacted with 5 history-taking chatbots. The chatbot configurations included 4 large language model (LLM) conditions in a 2×2 factorial design: detailed prompt with thinking mode (DT), detailed prompt with nonthinking mode (DN), minimal prompt with thinking mode (MT), and minimal prompt with nonthinking mode (MN), as well as a non-LLM chatbot baseline. Conversation transcripts were independently evaluated by physicians using a case-specific checklist. Information coverage was calculated and statistically compared across chatbot configurations.

An overview of the study workflow is presented in Figure 1. The chatbot prototype was developed with the primary aim of collecting patient medical histories prior to physician encounters, to support subsequent clinical documentation and decision-making.

The chatbot prototype was developed in Python (Python Software Foundation) using the Qwen3-14B-Instruct model as the core LLM. Qwen3-14B is an open-source, transformer-based model that demonstrates competitive reasoning and instruction-following capabilities while remaining computationally efficient for local deployment [29]. The model integrates “thinking” and “nonthinking” modes within a single framework, allowing flexible switching to examine how reasoning effort affects the completeness and interpretability of conversational outputs.

The system was implemented using Python 3.13.3, Streamlit 1.44.1 (Snowflake Inc), LangChain Core 0.3.51 (LangChain Inc), and LangChain Ollama 0.3.1 (LangChain Inc), with the Ollama backend managing model inference for the Qwen3-14B. Inference was conducted locally via Ollama, which handled model loading and execution without relying on external API calls. The conversational workflow was orchestrated through LangChain, which managed modular prompt structures and response generation. The Streamlit framework was used to build the graphical user interface, enabling real-time interaction with simulated patients (SPs) and structured data logging for subsequent analysis. Model inference used the following hyperparameters: temperature=0.6, top_*P*=.95, top_k=20, repeat_penalty=1, and num_predict=128.

#### Types of Comparison

We evaluated 4 LLM conditions in a 2×2 factorial design and compared them with an existing choice-based, rule-driven chatbot as a non-LLM baseline.

The arms were defined as follows: detailed prompt with thinking mode (DT), detailed prompt with nonthinking mode (DN), minimal prompt with thinking mode (MT), and minimal prompt with nonthinking mode (MN); choice, the baseline condition represented a conventional rule-based chatbot using predefined options without generative language modeling.

#### Operational Definitions

##### Prompt Design (Detailed vs Minimal)

The chatbot was guided by a detailed prompt, adapted from standard medical history-taking frameworks described in clinical textbooks [3,30], instructing it to act as a virtual medical assistant conducting comprehensive patient interviews. The prompt instructed the chatbot to generate only 1 question per response, avoid repeated questions, and maintain a smooth and natural conversation flow. It specified a structured interview sequence beginning with clarification of the reason for the visit and the main symptom, followed by symptom characterization, including onset, location, quality, severity, duration, triggers, relieving factors, recurrence, and prior treatments when clinically applicable. The chatbot was then instructed to ask about associated symptoms (ASs) across organ systems, to consider multiple differential diagnoses, to ask follow-up questions to support or rule out diagnostic possibilities, and to explore relevant external causes and risk factors. After sufficient information about the present illness had been gathered, the prompt instructed the chatbot to proceed in a fixed order to past medical history (PH), treatment and compliance, family history (FH), smoking and alcohol use, and conversation closure.

The minimal prompt instructed the chatbot to act as an AI-powered virtual medical assistant conducting a patient interview in English, generate 1 question per response, and to end the conversation when appropriate. It did not include explicit instructions regarding diagnostic reasoning, symptom characterization, AS, history-taking sequence, risk factors, or domain transitions.

The full text of both prompts is provided in Multimedia Appendix 1.

##### Internal Reasoning Configuration (Thinking vs Nonthinking)

In this study, “internal reasoning” refers to the generation of intermediate reasoning steps during response generation and was operationalized using the model’s built-in “thinking mode.” In this mode, complex, multistep deliberation occurred as intermediate reasoning steps before the model produced the final response, which were intended to guide response generation [29]. In practical terms, this configuration was expected to influence how the model selected follow-up questions and organized the next conversational step during the interview. The model’s internal reasoning process was configured in one of the two ways: (1) in the thinking mode, the model internally generated intermediate reasoning steps, while the user interface displayed only the final questions or responses; or (2) in the nonthinking mode, in which internal reasoning was disabled via a prompt instruction (/no_think), and questions were generated directly without intermediate deliberation.

### Baseline Description

Choice baseline is a commercially available symptom checker operating in Japan (Ubie, Inc) that guides users through fixed, multiple-choice questions based on branching clinical logic. This system is representative of current rule-based history-taking tools. Ubie was accessed in its English version through the publicly available web interface at the time of the study (which was operating in August 2025) [31].

### End-of-Conversation Determination

Each LLM chatbot interaction was designed to terminate under specific conditions reflecting the intended prompt structure and conversation control strategy. The termination criteria differed across configurations to reflect the varying degrees of reasoning autonomy and prompt guidance.

#### Detailed Prompt (End When Sufficient Information Is Obtained)

For chatbots provided with detailed prompts, conversation termination was guided by an instruction to conclude the interview after sufficient information had been collected. Specifically, the prompt instructed the chatbot to politely inform the patient that all necessary information had been collected, thank them for their cooperation, let them know they would see the physician next, and ask about their expectations or any other questions they wanted to ask the physician. This instruction was intended to guide the chatbot to end the information-gathering phase after completing the structured history-taking sequence.

#### Minimal Prompt (End When Appropriate)

In this mode, the chatbot autonomously decided when sufficient information had been gathered. The conversation ended once the system determined that the patient’s responses provided adequate information without explicit external cues.

#### Force-End Condition

Regardless of the configuration, all conversations were capped at 30 total messages (15 turns). This limit was selected to approximate a feasible preconsultation history-taking interaction while balancing information completeness with interaction burden for SPs. It also served to prevent excessively long or repetitive conversations. The same turn limit was applied to all LLM configurations to ensure comparability across modes. If this limit was reached before a natural conclusion, the conversation was automatically terminated, and the final message was recorded as the endpoint. The chatbot was not explicitly informed of this turn limit in the prompt; rather, the limit was implemented externally by the system.

This setup incorporated both prompt-guided and model-driven termination strategies to accommodate different levels of conversational control and ensure consistent stopping conditions across configurations.

### Case and Checklist

#### Case Selection

Symptom selection was guided by 3 reference sources covering primary care curricula from different countries, which were chosen to capture common clinical presentations encountered in general practice: the Royal College of General Practitioners curriculum [32], the Japanese Primary Care curriculum [33], and the symptom list is from the textbook *Primary Care: A Case-Based Office Evaluation* [34].

Two distinct clinical cases were created or selected for each symptom, each corresponding to a different final diagnosis. This resulted in a total of 66 standardized cases. Case selection prioritized clinical variety while maintaining presentation patterns representative of outpatient encounters.

The cases were primarily adapted from 4 core textbooks emphasizing case-based reasoning in general and family medicine: *100 Cases in Clinical Medicine* [35], *Bates’ Guide to Physical Examination and History Taking: Case Studies* [36], *The Patient History: An Evidence-Based Approach* [30], and *Case Files: Family Medicine* [37]

When suitable examples were insufficient in the selected textbooks, supplementary cases were adapted from journal case reports or published case studies, yielding a total of 9 cases [38-46]. All cases were reviewed by physicians to ensure that they contained the key elements of history-taking typically expected of a clinician when making a diagnosis. See Multimedia Appendix 1 for the complete list of 33 target symptoms and 2 representative diagnoses for each symptom. Each case was further summarized into a structured checklist for the evaluation of chatbot outputs.

#### Checklist Creation

A structured checklist was developed to assess whether the chatbot asked clinically relevant questions for each SP case. The checklist was designed to capture the completeness and clinical appropriateness of the chatbot’s biomedical inquiry, based on typical history-taking expectations in primary care.

Each checklist was organized into six domains as follows:

1. History of presenting complaint (HPC): onset, location, duration, character or quality, severity, progression, aggravating and relieving factors, treatments and their effectiveness, and any related trauma or accidents.
2. ASs: other symptoms mentioned in the case, whether present or specifically denied.
3. PH: existing conditions, comorbidities, and ongoing treatments.
4. FH: familial diseases or hereditary risks.
5. Social history (SH): history of smoking, alcohol consumption, lifestyle, and occupation
6. Other contextual information: additional relevant findings or case-specific details, including elaboration on AS when applicable.

Two physicians (a general practitioner and a family medicine resident) independently extracted checklist components from each reference case following a written protocol. The protocol specified that items were to be marked only if explicitly or implicitly mentioned in the case description, ensuring per case standardization rather than reliance on general expectations of history-taking. Discrepancies were reviewed, and final decisions were reached by consensus. An example of the created checklists is provided in Multimedia Appendix 1.

### Settings

The study was conducted in a controlled simulation environment using 6 SPs.

Six Thai nonmedical SPs with advanced proficiency in conversational English participated in the simulations. Each SP was assigned a fixed subset of 11 clinical cases, and each assigned case was portrayed by the same SP across all chatbot configurations. This approach was used to maintain consistent case portrayal across configurations while distributing the workload among SPs. Before data collection, SPs received standardized written instructions and a short orientation session. They were instructed to read the assigned case scenarios carefully, respond strictly in accordance with the provided case information, and avoid adding information not included in the scenario. For questions not explicitly addressed in the case script, SPs were instructed to avoid unsupported improvisation and to use “unknown/not specified” as the default response. If an “unknown/not specified” response seemed clinically implausible or inconsistent with the assigned case scenario, SPs were instructed to consult the researcher before responding. In such cases, the researcher confirmed whether the SP should use the default “unknown/not specified” response or provide a brief answer consistent with the general clinical features of the assigned case, without adding new case-specific information.

To reduce order effects, the order of chatbot configurations was randomized using a balanced rotation scheme generated by the researcher. The rotation was designed so that each configuration appeared in different ordinal positions across cases. SPs were not informed of the chatbot configuration labels during the simulations.

All simulations were performed locally on a MacBook Pro (Apple M4 Max, 128 GB of unified memory) to ensure consistent performance across all chatbot configurations.

Each clinical case was run once for each chatbot configuration, resulting in one transcript per case-configuration pair.

### Evaluation

#### Primary Evaluation

All chatbot-SP conversations were exported as structured text logs for independent physician evaluation. For the choice baseline, each fixed multiple-choice interaction was converted into a chronological text transcript containing the system questions, available response options, and the SP-selected option. The selected response was explicitly marked, and the resulting transcript was evaluated using the same case-specific checklist used for the LLM transcripts.

Before physician evaluation, each transcript was saved using a randomly assigned 6-digit transcript ID. The correspondence among the transcript ID, chatbot configuration, and case number was stored separately and not provided to physician raters. Therefore, physician raters were blinded to the LLM configuration labels during transcript review. However, the choice baseline could not be fully blinded because its fixed multiple-choice interaction format differed visibly from the open-ended LLM transcripts. This limitation was considered when interpreting comparisons involving the choice baseline.

Three physicians with prior OSCE evaluation experience independently reviewed the transcripts to assess coverage completeness, defined as the proportion of items on from the reference checklist that were appropriately elicited by the chatbot.

Each checklist item was marked as covered or not covered. When disagreements occurred, the final score for each item was determined by majority consensus (2 of the 3 raters). Coverage percentage for each case was then calculated as:


$$\text{Coverage}\;(\%)=\frac{\text{Number of covered items}}{\text{Total items in checklist}}\times 100$$

For each chatbot configuration, coverage percentages were macroaveraged across the 66 cases.

#### Subgroup Analysis by Question Type

Coverage was further examined by question type, corresponding to the checklist domains of HPC, ASs, PH, FH, and SH.

For the subgroup analysis by question type, each domain-specific analysis included only cases containing at least one checklist item in that domain. Therefore, the number of cases differed across domains because not all reference cases included PH, FH, SH, or AS items. Domain-specific coverage was calculated as the number of covered items divided by the total number of checklist items within that domain for each eligible case. The mean and SD of these case-level coverage percentages were then calculated for each chatbot mode within each domain.

#### Symptom-Based Analysis

To explore variation across clinical presentations, coverage results were further summarized by symptom. We identified the highest and lowest macroaverage coverage percentages per symptom across all modes, and the largest advantage (Δ coverage) of the DT mode compared with the average of the other 3 LLM configurations (DN, MT, and MN).

This approach allowed the examination of symptom-level performance patterns and the identification of clinical scenarios in which the combination of detailed prompting and the thinking mode had the greatest impact.

#### Repetitive Questioning Analysis

To further characterize chatbot behavior beyond checklist coverage, 1 researcher conducted an exploratory descriptive review of repetitive questioning in the 4 open-ended LLM configurations. Repetitive questioning was defined as asking the same or substantially similar questions more than once despite a clear prior answer from the SP. Repeated questioning was not counted when the SP had not clearly answered the previous question, such as when a multipart question was only partially answered. This analysis was descriptive and exploratory.

#### Interrater Agreement

Agreement among the three physician evaluators on item-level coverage was assessed using 2 complementary metrics: (1) the proportion of items with full agreement across all raters and (2) Fleiss κ, calculated with the *statsmodels* library in Python (version 3.13.3).

### Statistical Analysis

To formally examine the factorial effects among the 4 LLM configurations, a 2-way repeated-measures ANOVA was conducted with prompt detail and internal reasoning as within-case factors. The choice chatbot was excluded from this factorial analysis. To compare all evaluated configurations, including the choice baseline, a 1-way repeated-measures ANOVA was additionally conducted across the 5 configurations, followed by post hoc 2-tailed paired *t* tests with Bonferroni correction. Sphericity was assessed using the Mauchly test, and for repeated-measures ANOVA with more than 2 levels, Greenhouse–Geisser-corrected *P*-values were used when the sphericity assumption was violated. Effect sizes reported for the factorial 2-way repeated-measures ANOVA and the overall 1-way repeated-measures ANOVA were expressed as generalized eta squared (η²_G_), and pairwise effect sizes were reported as Hedge *g*.

All analyses were conducted in a Jupyter Notebook (Project Jupyter) using Python 3.13.3 and *Pingouin* package, with statistical significance set at *P*<.05.

### Ethical Considerations

This study was considered exempt from formal ethics review under the Ethical Guidelines for Medical and Biological Research Involving Human Subjects in Japan [47] because it used standardized fictional or publicly available case scenarios that did not contain information relating to identifiable individuals and because it involved SPs who were nonmedical volunteers recruited outside clinical care settings and who role-played predefined cases without providing personal health information. All SPs were informed of the study’s purpose and procedures before participating and agreed to participate voluntarily. Transcript data were labeled using randomly assigned study IDs, and no personally identifiable information was included in the evaluation files, figures, or supplementary materials. SPs received a small honorarium for their participation. No identifiable participant images or personal information is included in the manuscript or supplementary materials.

## Results

### Interrater Agreement

To assess the reliability of grading across evaluators, interrater agreement was calculated based on all checklist items. The evaluators achieved an overall full agreement rate of 81.90%, indicating a high level of consistency in their assessments. The Fleiss κ value was 0.753, representing substantial agreement according to the Landis and Koch Benchmark Scale [48]. These results suggest that the evaluation criteria were well-defined and consistently applied among raters.

### Comparison of Coverage Across Chatbot Modes

Across all 66 SP cases, coverage rates varied substantially among the 5 chatbot configurations (Table 1). The DT mode achieved the highest mean coverage (72.3%, SD 14.3%), followed by the MT mode (60.5%, SD 15.0%), the DN mode (59.8%, SD 13.6%), the MN mode (54.0%, SD 15.3%), and the choice chatbot (51.1%, SD 13.3%).

To formally examine the factorial effects among the 4 LLM configurations, a 2-way repeated-measures ANOVA was conducted with prompt detail and internal reasoning as within-case factors. This analysis showed significant main effects of prompt detail (*F*_1, 65_=56.74; *P*<.001; η²_G_=0.085) and internal reasoning (*F*_1, 65_=47.45; *P*<.001; η²_G_=0.096). A significant interaction between prompt detail and internal reasoning was also observed (*F*_1, 65_=6.32; *P*=.01; η²_G_=0.011), indicating that the effect of internal reasoning differed according to prompt detail. The DT configuration achieved the highest overall coverage among the 4 LLM configurations.

To compare all evaluated configurations, including the choice baseline, a 1-way repeated-measures ANOVA was additionally conducted across the 5 configurations. Sphericity was assessed using Mauchly test and was not violated. Therefore, uncorrected results are reported. This analysis revealed a significant effect of chatbot mode on coverage (*F*_4, 260_=36.88; *P*<.001; η²_G_=0.21), indicating that the completeness of gathered information differed significantly across chatbot modes.

Post hoc pairwise *t* tests with Bonferroni correction (Table 2) showed that the DT mode significantly outperformed all other configurations. Among the remaining configurations, DN and MT did not differ significantly (*P*>.99). However, both DN and MT achieved higher coverage than MN and choice. The MN and choice modes did not differ significantly (*P*>.99).

Overall, these findings demonstrate that both prompt detail and internal reasoning are associated with improved information completeness, with their combination in the DT mode producing the greatest coverage.

**Table 1.** Mean coverage percentages (SD) across 5 chatbot configurations in 66 simulated patient interviews. Each configuration represents a combination of prompt detail (detailed vs minimal) and internal reasoning (thinking vs nonthinking).

Chatbot mode	Prompt type	Reasoning mode	Average coverage (%), mean (SD)	
DT^a^	Detailed	Thinking	72.3 (14.3)	
DN^b^	Detailed	Nonthinking	59.8 (13.6)	
MT^c^	Minimal	Thinking	60.5 (15.0)	
MN^d^	Minimal	Nonthinking	54.0 (15.3)	
Choice			51.1 (13.3)	

a DT: detailed prompt with thinking mode.

b DN: detailed prompt with nonthinking mode.

c MT: minimal prompt with thinking mode.

d MN: minimal prompt with nonthinking mode.

**Table 2.** Post hoc pairwise *t* test comparisons between chatbot configurations using a repeated-measures design. Bonferroni correction was applied for multiple comparisons.

Comparison (A)	Comparison (B)	*2-tailed t* test (*df*)	*P* (Bonferroni-corrected)	Hedge *g*
DT^a^	DN^b^	7.20 (65)	<.001	0.88
DT	MT^c^	7.05 (65)	<.001	0.80
DT	MN^d^	9.40 (65)	<.001	1.22
DT	Choice	9.71 (65)	<.001	1.53
DN	MT	−0.39 (65)	>.99	−0.05
DN	MN	3.50 (65)	.008	0.40
DN	Choice	4.52 (65)	<.001	0.65
MT	MN	3.41 (65)	.01	0.42
MT	Choice	4.31 (65)	<.001	0.66
MN	Choice	1.48 (65)	>.99	0.21

a DT: detailed prompt with thinking mode.

b DN: detailed prompt with nonthinking mode.

c MT: minimal prompt with thinking mode.

d MN: minimal prompt with nonthinking mode.

### Subgroup Analysis by Question Type

Coverage was further examined across 5 question types based on mean coverage: HPC, ASs, PH, FH, and SH (Table 3). The DT mode achieved the highest coverage in most question categories, including HPC, AS, FH, and SH; however, the choice baseline showed slightly higher coverage for PH. The DN and MT modes showed intermediate performance, while MN and the choice chatbot demonstrated the lowest coverage overall, except for the high PH coverage observed in the choice baseline.

Marked variation was observed across question types: HPC and AS achieved relatively high coverage in most modes, whereas PH, FH, and SH showed greater discrepancies among configurations.

A series of 1-way repeated-measures ANOVAs revealed significant differences in coverage among chatbot modes for all question types. Sphericity was assessed using Mauchly test, and Greenhouse-Geisser-corrected *P*-values were used when sphericity was violated. Significant differences were observed for all question types: HPC (n=66; *F*_4, 260_=12.06; *P*<.001), AS (n=64; *F*_4, 252_=7.69; *P*<.001), PH (n=57; *F*_4, 224_=34.39; *P*<.001), FH (n=28; *F*_4, 108_=50.12; *P*<.001), and SH (n=57; *F*_4, 224_=13.47; *P*<.001).

Significant mode-related differences were consistent across all question types, with the largest effects observed for FH and PH.

**Table 3.** Mean coverage percentages (SD) for each chatbot configuration by question type^a^.

Question type	Cases, n	DT (%), mean (SD)^b^	DN (%), mean (SD)^c^	MT (%), mean (SD)^d^	MN (%), mean (SD)^e^	Choice (%), mean (SD)
HPC^f^	66	79.0 (19.7)	77.3 (20.2)	69.5 (26.0)	60.4 (28.7)	62.6 (22.0)
AS^g^	64	80.1 (22.9)	76.0 (22.5)	78.7 (25.0)	75.8 (23.2)	64.7 (26.2)
PH^h^	57	93.0 (19.9)	36.0 (48.0)	53.5 (48.1)	45.6 (46.6)	94.7 (15.5)
FH^i^	28	98.2 (9.4)	7.1 (26.2)	58.9 (49.2)	21.4 (41.8)	3.6 (18.9)
SH^j^	57	45.3 (38.4)	12.0 (25.7)	19.3 (31.8)	21.1 (35.1)	11.4 (27.3)

a Sample sizes differ because the analyses were restricted to cases containing at least one checklist item in each domain.

b DT: detailed prompt with thinking mode.

c DN: detailed prompt with nonthinking mode.

d MT: minimal prompt with thinking mode.

e MN: minimal prompt with nonthinking mode.

f HPC: history of presenting complaint.

g AS: associated symptom.

h PH: past medical history.

i FH: family history.

j SH: social history.

### Symptom-Based Analysis

The highest and lowest macroaveraged coverage percentages per symptom across all modes were examined. To explore variation across clinical presentations, coverage was further summarized by symptoms (Table 4).

**Table 4.** Top 3 and bottom 3 symptoms ranked by mean macroaverage coverage across all chatbot modes (n=33 symptoms).

Rank	Symptom	Average coverage (%), mean (SD)
Highest coverage		
1	Palpitations	77.60 (14.50)
2	Joint pain	75.23 (6.77)
3	Abdominal pain	72.83 (8.78)
Lowest coverage		
31	Fever	47.79 (9.13)
32	Impaired vision	46.11 (5.76)
33	Nausea and vomiting	46.07 (6.34)

Across all chatbot modes, the symptoms with the highest average coverage were palpitations (77.60%, SD 14.50%), joint pain (75.23%, SD 6.77%), and abdominal pain (72.83%, SD 8.78%).

In contrast, the lowest-coverage symptoms were nausea and vomiting (46.07%, SD 6.34%), impaired vision (46.11%, SD 5.76%), and fever (47.79%, SD 9.13%).

These results highlight substantial variation in information completeness across presenting complaints.

The full list of 33 symptoms is provided in Multimedia Appendix 1

We then identified the largest advantage (Δ coverage) of the DT mode over the average of the other 3 LLM configurations (DN, MT, and MN).

To identify the symptom types with the greatest improvement under the DT configuration, we calculated the difference in mean coverage between DT and the average of the other 3 LLM modes (DN, MT, and MN).

The 5 symptoms showing the largest relative advantage of DT were weakness, headache, dysphagia, palpitations, and dizziness. Among these 5 symptoms with the largest DT advantage, coverage improvements ranged from approximately 22.4%-30.6% points compared with the average of the other LLM configurations. Pairwise differences between DT and individual LLM configurations are shown in Table 5.

**Table 5.** Top 5 symptoms showing the largest relative advantage of the detailed prompt with the thinking mode (DT) configuration in coverage percentage compared with other large language model (LLM) configurations.

Symptom	Δ coverage (DT^a^ - Mean of DN, MT, and MN) (%)	DT-DN^b^ (%)	DT-MT^c^ (%)	DT-MN^d^ (%)
Weakness	30.6	30.3	21.2	40.4
Headache	30.0	30.0	35.0	25.0
Dysphagia	28.2	25.8	14.6	44.2
Palpitations	26.8	33.2	15.4	31.7
Dizziness	22.4	22.6	17.9	26.8

a DT: detailed prompt with thinking mode.

b DN: detailed prompt with nonthinking mode.

c MT: minimal prompt with thinking mode.

d MN: minimal prompt with nonthinking mode.

### Repetitive Questioning Analysis

In an exploratory review of repetitive questioning, repeated questions were observed in 4 of 66 (6.1%) DT transcripts, 7 of 66 (10.6%) DN transcripts, 3 of 66 (4.5%) MT transcripts, and 10 of 66 (15.2%) MN transcripts. Repetitive questioning was uncommon overall but occurred more often in nonthinking configurations than in thinking configurations, most frequently in the MN configuration.

## Discussion

### Principal Findings

This study demonstrates that LLM-based chatbot interviews are capable of collecting patient-reported medical history across multiple clinical areas in a controlled, simulated setting. Across the evaluated settings, LLM-based chatbots achieved overall information coverage comparable to or exceeding that of the existing choice-based system, supporting their feasibility as flexible tools for collecting baseline patient history. Notably, the MN configuration performed at a level comparable to the choice-based baseline. This indicates that the use of an LLM alone, without structured prompting or reasoning support, does not inherently provide an advantage. Instead, the completeness and clinical relevance of the collected information, as reflected by case-based checklist coverage, were strongly influenced by prompt design and the presence of internal reasoning mechanisms.

Among the evaluated configurations, the DT mode achieved the highest overall coverage. Compared with other LLM configurations and the choice-based baseline, DT achieved an absolute improvement in information coverage of approximately 12‐21 percentage points at a fixed interaction length. The factorial analysis showed additional significant main effects of prompt detail and internal reasoning, as well as a significant interaction between prompt detail and internal reasoning. These findings suggest that detailed prompting and internal reasoning each contributed to improved coverage, and that their combination was associated with the greatest information capture in this controlled simulated setting.

Prompt detail appeared to play an important role in establishing a clinically coherent interview structure. While the underlying language model could collect relevant patient information, detailed prompts improved the consistency and depth of history taking. Configurations lacking sufficient prompt structure tended to produce less detailed information in several history components, resulting in an overall reduction in coverage. These findings suggest that prompt detail functions as a clinical framework, aligning the model’s flexible behavior with established medical interviewing frameworks and supporting more reliable information collection.

Internal reasoning mechanisms may have further contributed by enabling prioritization and adaptive follow-up questioning. When internal reasoning was absent, chatbots tended to remain within the same history domain that was emphasized early in the prompt, resulting in limited progression to subsequent components. In contrast, reasoning-enabled configurations appeared better able to identify clinically relevant information and pursue follow-up questions that extended beyond the initial domain, such as PH, FH, and SH. These findings suggest that internal reasoning may support dynamic decision-making within the interview, complementing structured prompts by guiding when and how to transition between history components. In the exploratory review of repetitive questioning, repeated questions were observed slightly more often in nonthinking configurations than in thinking configurations. This pattern may suggest that internal reasoning helped the chatbot make more consistent use of prior conversational context in some cases. However, because the internal reasoning process itself was not directly analyzed, this interpretation should be considered a possible explanation rather than a confirmed mechanism. Further qualitative error analysis of transcript-level behavior would be needed to better examine how reasoning-mode configurations influence question selection, domain transitions, and use of prior conversational context.

The low coverage observed in SH, even in the DT configuration, should be interpreted in relation to the alignment between the prompt’s scope and the checklist criteria. To prevent the chatbot from drifting into overly broad or nonclinical lifestyle discussions, social history questioning was limited to key clinically relevant risk factors, specifically smoking and alcohol consumption. Consequently, lower SH coverage may partly reflect a mismatch between the prompt’s scope and the grading checklist, rather than a general inability of the chatbot to collect social history. While this design choice supported a focused interview, it resulted in limited coverage of other SH subcategories included in the grading checklist that were not explicitly prompted. This limitation reflects broader challenges in capturing social history information, as SH sections can contain a wide range of social, behavioral, and environmental determinants, yet they are variably documented in practice [49]. In real-world use, the scope of social history questioning may need to be adapted according to the clinical scenario, specialty, or intended use of the chatbot in clinical care.

Performance varied across clinical scenarios. Symptoms linked to specific organ systems and common diagnostic pathways, such as palpitations, joint pain, and abdominal pain, were associated with higher coverage, whereas systemic or nonspecific presentations, such as fever and nausea and vomiting, were more challenging because they may require broader and less linear exploration across multiple organ systems. This suggests that a single static interviewing strategy is unlikely to perform optimally across all contexts and that adaptation to the presenting complaint may be required. The largest gains with the DT mode were observed in cases containing distinctive diagnostic cues (eg, transient visual loss in suspected transient ischemic attack or thunderclap onset in subarachnoid hemorrhage). In these scenarios, the combination of detailed prompting and internal reasoning may have helped the chatbot ask more targeted follow-up questions, although this mechanism was not directly tested.

Taken together, these results suggest that chatbot-based systems may be capable of collecting clinically relevant patient history information in controlled, simulated settings, supporting their potential role in preconsultation data gathering. The findings further suggest that the reliability and completeness of this information depend on the integration of multiple design elements, including sufficiently detailed prompts to provide clinical structure and internal reasoning mechanisms to support prioritization and progression through the interview. In practice, such systems are best positioned as supportive tools that augment baseline information collection alongside clinician-led interviews.

### Comparison With Prior Work

Consistent with previous studies [16,26], our results support the feasibility of using chatbots to collect patient medical histories prior to physician encounters. Beyond demonstrating feasibility, this study extends prior work by quantitatively evaluating information coverage across multiple clinical domains and symptom types. The findings further suggest that structured prompt design and the inclusion of internal reasoning mechanisms, as explored in earlier studies [22,25], can support clinically oriented questioning, leading to more comprehensive and diagnostically relevant information collection.

### Limitations

This study has several limitations. First, the fixed 15-turn limit may have truncated deeper questioning, potentially underestimating the full capabilities of the chatbot configurations, particularly for later history domains such as PH, FH, and social history. Because this limit was implemented externally and was not explicitly stated in the chatbot prompts, the models could not anticipate the remaining number of turns when prioritizing questions. Lower coverage in these later domains may therefore partly reflect the predefined conversation-length limit rather than the model’s full potential capabilities. In addition, this study did not formally analyze how conversation length or stopping point affected performance.

Second, the results are based solely on the Qwen3-14B-Instruct model. While this model has demonstrated strong reasoning capabilities, the findings regarding “thinking mode” may vary across different model architectures or larger-scale models.

Third, only a single detailed prompt design was evaluated, and the results may not generalize to other prompt styles or instructional formulations.

Fourth, the symptom-level analyses were based on a limited number of simulated cases (2 cases per symptom), which may not fully capture the heterogeneity of real-world clinical presentations.

Fifth, the evaluation was conducted using SP cases in a controlled setting, and chatbot performance may differ in real-world clinical environments.

Sixth, the low SH coverage should be interpreted as a limitation in prompt-rubric alignment. It may partly reflect the evaluation design rather than an inherent inability of the model to collect social history information.

Seventh, the choice chatbot differed from the LLM configurations in its design objectives, interface format, branching logic, and stopping rules. Therefore, it should be interpreted as a pragmatic baseline reference rather than a fully equivalent comparator. In addition, although physician raters were blinded to LLM configuration labels, the choice baseline could not be fully blinded during evaluation because its fixed multiple-choice format was visibly different from the open-ended format of the LLM transcripts.

Finally, the outcome in this study was checklist-based information coverage, which remains a surrogate measure. Because the checklists were derived from standardized case information, the coverage score primarily reflected whether the chatbot elicited expected case-specific information, rather than whether it performed an optimal or fully individualized clinical interview. Higher coverage does not necessarily indicate better diagnostic accuracy, clinical safety, patient experience, conversational efficiency, or real-world usefulness.

### Future Directions

Future work should evaluate chatbot performance in real clinical settings, where patient perceptions, communication styles, and time constraints vary more widely. Integrating chatbot-collected histories with clinician documentation workflows and examining the impact of this integration on documentation quality and patient-physician information alignment will be important next steps. Future studies should also examine how chatbot-collected histories can be summarized and presented to clinicians, whether findings generalize to other LLMs, including larger frontier models, and how conversation length, adaptive stopping strategies, and transcript-level error patterns, such as repetitive questioning, missed transitions, and irrelevant follow-up questions, influence information coverage and conversational quality. Future simulation-based studies should also consider analytical approaches that account for simulated-patient-level variability, as well as clinical safety outcomes, such as hallucination-related errors and potentially unsafe responses.

### Conclusions

This study evaluated the effects of prompt detail and internal reasoning on checklist-based information coverage in LLM-based chatbot medical history taking using standardized primary care cases. In this controlled simulated setting, the LLM chatbot was able to collect patient history information across multiple clinical domains, with the detailed prompt and thinking mode achieving the highest overall coverage among the evaluated configurations. The results indicate that both structured clinical prompt design and internal reasoning configuration influence the completeness of chatbot-collected patient history information, with performance varying across symptom types. These findings support the feasibility of LLM-based chatbots for preconsultation history collection within the scope of checklist-based evaluation in a controlled simulated setting.

## Supplementary material

10.2196/94614 Multimedia Appendix 1 Supplementary materials comprising the full detailed prompt (Section A), the complete list of 33 target symptoms, and 2 representative diagnoses for each symptom (Section B), an example of a case-specific checklist developed for evaluation (Section C), and the full ranking of the 33 symptoms by macroaverage coverage percentage across all chatbot modes (Section D).

## Abbreviations

AS: associated symptom
DN: detailed prompt with nonthinking mode
DT: detailed prompt with thinking mode
FH: family history
HPC: history of presenting complaint
LLM: large language model
MN: minimal prompt with nonthinking mode
MT: minimal prompt with thinking mode
OSCE: Objective Structured Clinical Examination
PH: past medical history
SH: social history
SP: simulated patient
