# Supplementary material for: Evaluation of Prompt Design and Internal Reasoning in Chatbot-Based Medical History Taking: Simulation Study
Source: JMIR Med Inform. 2026 Aug 21;14:e94614. doi: 10.2196/94614 (PMC13501399; doi:10.2196/94614)
Supplement: Supplementary file 1 [file medinform-v14-e94614-s001.docx]

## Section A: Full detailed prompt

### Detailed prompt

"""You are an AI-powered virtual medical assistant conducting a patient interview in English.

Your task is to gather detailed information about the patient’s symptoms and think through possible diagnoses to guide your questioning.

Instruction:

- Generate only the question content.

- Output only **one** question per response in English.

- Avoid repeating questions that have already been asked, **unless the patient has not answered yet** or the response was unclear.

- Ensure that the flow of conversation is smooth and natural, avoiding abrupt topic shifts or disorganized questioning.

- Think efficiently: Only perform as much reasoning as needed to decide on the next best question. If the next question is obvious, skip deeper analysis and ask it directly.

Think step by step—but be flexible and efficient:

1. First, determine why the patient came today.

  - If the patient came for a screening or health check-up and does **not report any symptoms**, politely thank the patient and end the conversation by letting them know they will next meet with the physician.

  - If the patient has any symptoms, proceed with a detailed interview.

2. Identify and clarify the main symptom if present.

3. Before proceeding to associated symptoms, gather relevant details for each reported symptom, but only if clinically meaningful or applicable. Focus on the following aspects when appropriate:

  - Onset

  - Location

  - Quality (e.g., sharp, dull, throbbing)

  - Severity

  - Duration and course

  - Triggers and relieving factors

  - Recurrence history

  - Past treatments and their effects

4. After fully exploring each main symptom, ask about associated symptoms from multiple organ systems—especially those that help narrow the differential diagnosis or suggest serious conditions.

5. As you collect information, actively consider multiple possible differential diagnoses. Do not stop at just one explanation. Stay broad and open in your reasoning.

6. Ask follow-up questions that help support or rule out each diagnostic possibility based on the evolving context.

7. Explore external or environmental causes (e.g., trauma, infection exposure, allergens) as needed.

8. Ask about relevant risk factors (e.g., lifestyle, exposures, comorbidities) when applicable to the suspected conditions.

9. Ensure that sufficient information about the current symptoms, associated symptoms and past treatment (History of Present Illness) is gathered. Then proceed to ask about the following in this fixed order:

  - Past medical history, treatment, and compliance

  - Family history

  - Smoking and alcohol use

  - Then conclude the conversation by politely informing the patient that all necessary information has been collected, thank them for their cooperation, and let them know they will next see the doctor. Ask about expectations and other questions they want to ask the physician.

Patient Information:

- Responder: {responder}

- Age: {age} years old

- Gender: {gender}

Patient Interview Transcript:

{conversation_history}

"""

### Minimal prompt

"""You are an AI-powered virtual medical assistant conducting a patient interview in English.

Instruction:

- Output only **one** question per response in English.

- End the conversation when appropriate.

Patient Information:

- Responder: {responder}

- Age: {age} years old

- Gender: {gender}

Patient Interview Transcript:

{conversation_history}

"""

## Section B:  complete list of 33 target symptoms and two representative diagnoses for each symptom

1. Chest Pain: myocardial infarction, anxiety
2. Dyspnea: chronic obstructive pulmonary disease, pulmonary embolism
3. Edema: chronic venous insufficiency, diabetic nephropathy
4. Palpitations: atrial fibrillation, ventricular tachycardia
5. Loss of consciousness: seizure, vasovagal syncope
6. Rash: systemic lupus erythematosus, tinea corporis
7. Hearing loss: presbycusis, noise-induced hearing loss
8. Epistaxis: Wegener's granulomatosis, anterior epistaxis
9. Hoarseness: vocal cord paralysis, fungal laryngitis
10. Nasal obstruction: allergic rhinitis, common cold
11. Sore throat: streptococcal pharyngitis, infectious mononucleosis
12. Jaundice: acute hepatitis A, pancreatic adenocarcinoma
13. Weight loss: anorexia, colon cancer
14. Red eye: glaucoma, viral conjunctivitis
15. Impaired vision: multiple sclerosis with optic neuritis, Compressive III nerve palsy from an aneurysm
16. Abdominal pain: acute cholecystitis, diverticulitis
17. Bowel habit change: ulcerative colitis, irritable bowel syndrome
18. Dyspepsia: gastroesophageal reflux disease (different scenarios)
19. Dysphagia: esophageal ring, achalasia
20. Nausea vomiting: peptic ulcer with partial gastric outlet obstruction, cannabinoid Hyperemesis Syndrome
21. Gastrointestinal bleeding: variceal bleeding, diverticulosis
22. Breast mass: breast cyst, breast cancer
23. Abnormal vaginal bleed: polycystic ovarian syndrome, uterine fibroids
24. Fever: sinusitis, acute retroviral syndrome
25. Hematuria: autosomal dominant polycystic kidney disease, urinary tract infection
26. Joint pain: rheumatoid arthritis, septic arthritis
27. Cognitive impairment: delirium from dehydration and urosepsis, dementia
28. Cough: asthma, lobar pneumonia
29. Fatigue: pernicious anemia, hyperthyroidism
30. Headache: subarachnoid hemorrhage, migraine without aura
31. Dizziness: Intermittent heart block, benign paroxysmal positional vertigo
32. Back pain: tumor metastasis, lumbar sacral strain
33. Weakness: transient ischemic attack, Guillain-Barré syndrome

## Section C: Example of checklist developed from case

| Components | Transcript ID_1 | Transcript ID_2 | Transcript ID_3 | Transcript ID_4 | Choice | Expected Answer from SP |
| --- | --- | --- | --- | --- | --- | --- |
| History of Presenting Complaint |  |  |  |  |  |  |
| Was **Onset** asked? |  |  |  |  |  | 1 hour ago |
| Was **Location** asked? |  |  |  |  |  | under the sternum |
| Was **Characteristics** asked? |  |  |  |  |  | pressure |
| Was **Severity** or **Impact on Daily Life** asked? |  |  |  |  |  | 7/10 in intensity |
| Was **Radiation** asked? |  |  |  |  |  | radiates into his left arm and up into his jaw |
| Was **Aggravating factor** asked? |  |  |  |  |  | climbing the stairs |
| Was **Alleviating factor** asked? |  |  |  |  |  | rest |
| Was **Past treatment** and **effectiveness** asked? |  |  |  |  |  | sublingual nitroglycerin helps ease the pain |
| Was **Past episodes / Recurrence** asked? |  |  |  |  |  | had similar symptoms during the last 2 weeks, but these episodes have lasted for 5 to 10 minutes at the most. |
| Associated Symptoms |  |  |  |  |  |  |
| Was **shortness of breath** asked? |  |  |  |  |  | Present |
| Was **nausea** asked? |  |  |  |  |  | Present |
| Was **sweating** asked? |  |  |  |  |  | Present |
| Was **palpitations** asked? |  |  |  |  |  | Denied |
| Was **blurred vision** asked? |  |  |  |  |  | Denied |
| Was **vomiting** asked? |  |  |  |  |  | Denied |
| Was **claudication** asked? |  |  |  |  |  | Denied |
| Past Medical History |  |  |  |  |  |  |
| Was **past history** asked? |  |  |  |  |  | Hypertension (25 yrs), GERD (15 yrs). |
| Family History |  |  |  |  |  |  |
| Was **family history** asked? |  |  |  |  |  | Father died of MI at 50; paternal grandfather died of MI at 45; mother has hypertension. |
| Social history |  |  |  |  |  |  |
| Were **smoking and alcohol** asked? |  |  |  |  |  | Smokes 1 pack/day since age 15; drinks alcohol socially; denies illicit drug use. |
| Was **occupation-related factors** asked? |  |  |  |  |  | advertising executive |

## Section D: The full list of 33 symptoms ranked by macro-average coverage percentages per symptom across all modes

| Rank | Symptom | Average Coverage (%), mean (SD) |
| --- | --- | --- |
| 1 | Palpitations | 77.60 (14.50) |
| 2 | Joint pain | 75.23 (6.77) |
| 3 | Abdominal pain | 72.83 (8.78) |
| 4 | Loss of consciousness | 70.26 (11.50) |
| 5 | Nasal obstruction | 69.75 (12.79) |
| 6 | Hearing loss | 68.73 (12.23) |
| 7 | Edema | 67.82 (11.11) |
| 8 | Weakness | 64.55 (15.82) |
| 9 | Sore throat | 64.17 (7.49) |
| 10 | Gastrointestinal bleeding | 62.70 (10.49) |
| 11 | Chest Pain | 62.62 (6.87) |
| 12 | Weight loss | 62.19 (10.77) |
| 13 | Dyspepsia | 62.18 (14.90) |
| 14 | Dyspnea | 61.92 (3.91) |
| 15 | Jaundice | 61.67 (17.67) |
| 16 | Back pain | 60.51 (16.39) |
| 17 | Dizziness | 60.36 (10.22) |
| 18 | Headache | 58.00 (13.51) |
| 19 | Breast mass | 57.14 (11.45) |
| 20 | Fatigue | 56.92 (7.88) |
| 21 | Epistaxis | 56.67 (7.41) |
| 22 | Abnormal vaginal bleed | 54.61 (6.35) |
| 23 | Red eye | 54.40 (9.00) |
| 24 | Hematuria | 53.33 (14.63) |
| 25 | Cognitive impairment | 52.67 (12.07) |
| 26 | Cough | 52.62 (5.70) |
| 27 | Bowel habit change | 52.12 (10.76) |
| 28 | Hoarseness | 51.67 (8.92) |
| 29 | Dysphagia | 50.22 (21.15) |
| 30 | Rash | 49.43 (4.89) |
| 31 | Fever | 47.79 (9.13) |
| 32 | Impaired vision | 46.11 (5.76) |
| 33 | Nausea/vomiting | 46.07 (6.34) |
